# CAR Co-Operates With Integrins to Promote Lung Cancer Cell Adhesion and Invasion

**DOI:** 10.3389/fonc.2022.829313

**Published:** 2022-02-14

**Authors:** Claudia Owczarek, Elena Ortiz-Zapater, Jana Kim, Efthymia Papaevangelou, George Santis, Maddy Parsons

**Affiliations:** ^1^ Randall Centre for Cell and Molecular Biophysics, King’s College London, London, United Kingdom; ^2^ School of Biomedical Engineering and Imaging Sciences, King’s College London, St Thomas Hospital, London, United Kingdom; ^3^ Peter Gorer Department of Immunobiology, School of Immunology and Microbial Science, King’s College London, London, United Kingdom

**Keywords:** cell-cell adhesion, cell-matrix adhesion, invasion, integrins, coxsackievirus adenovirus receptor (CAR), lung cancer

## Abstract

The coxsackie and adenovirus receptor (CAR) is a member of the junctional adhesion molecule (JAM) family of adhesion receptors and is localised to epithelial cell tight and adherens junctions. CAR has been shown to be highly expressed in lung cancer where it is proposed to promote tumor growth and regulate epithelial mesenchymal transition (EMT), however the potential role of CAR in lung cancer metastasis remains poorly understood. To better understand the role of this receptor in tumor progression, we manipulated CAR expression in both epithelial-like and mesenchymal-like lung cancer cells. In both cases, CAR overexpression promoted tumor growth *in vivo* in immunocompetent mice and increased cell adhesion in the lung after intravenous injection without altering the EMT properties of each cell line. Overexpression of WTCAR resulted in increased invasion in 3D models and enhanced β1 integrin activity in both cell lines, and this was dependent on phosphorylation of the CAR cytoplasmic tail. Furthermore, phosphorylation of CAR was enhanced by substrate stiffness *in vitro*, and CAR expression increased at the boundary of solid tumors *in vivo*. Moreover, CAR formed a complex with the focal adhesion proteins Src, Focal Adhesion Kinase (FAK) and paxillin and promoted activation of the Guanine Triphosphate (GTP)-ase Ras-related Protein 1 (Rap1), which in turn mediated enhanced integrin activation. Taken together, our data demonstrate that CAR contributes to lung cancer metastasis *via* promotion of cell-matrix adhesion, providing new insight into co-operation between cell-cell and cell-matrix proteins that regulate different steps of tumorigenesis.

## Introduction

Lung cancer is the leading cause of cancer death worldwide (1). Despite advances in treatment options, prognosis remains poor as lung cancer is mainly diagnosed at advanced stages (2). To metastasise, tumor cells must undergo a multi-step process defined as the metastatic cascade. Metastasis requires cell detachment from the primary tumor, local invasion, intravasation and survival in the vasculature, extravasation, and colonisation of a secondary site (3). Adhesion molecules play a crucial role in all the metastatic steps as they regulate adhesive properties and integrate extracellular cues with cell intrinsic signalling to regulate other cellular functions (4). Numerous previous studies have suggested that metastasis requires cancer cells to undergo epithelial to mesenchymal transition (EMT) whereby cell-cell adhesions are downregulated to promote a more pro-migratory phenotype. However, recent studies have highlighted the dynamic nature of these phenotypic transitions. Indeed, recent studies discovered the existence of a wide spectrum of intermediate states whereby a mix of epithelial and mesenchymal markers are expressed (5).

Members of the Junctional Adhesion Molecule (JAM) family are aberrantly expressed in several types of cancer where they play a dual role. JAMs are type I transmembrane glycoproteins characterised by the presence of two extracellular immunoglobulin (Ig)-like domains and include JAM-A, JAM-B, JAM-C and Coxsackie and Adenovirus Receptor (CAR) (6). CAR was originally identified as a docking receptor for adenovirus type 2 and 5 and coxsackie B virus and subsequently recognized as a key regulator of cell-cell adhesion (7, 8). CAR is comprised of an extracellular domain (containing D1 and D2 domains), a transmembrane region and an unstructured cytoplasmic tail (9). CAR is found at both tight and adherens junctions where it regulates cell-cell adhesion *via* homodimerization *in trans* between CAR D1 domains present on adjacent cells and/or *via* cytoplasmic interactions with other adhesion molecules such as Zonula Occludens 1 (ZO-1), E-cadherin and β-catenin (10–13). Phosphorylation at Ser290/Thr293 residues in the CAR cytoplasmic tail by Protein Kinase C δ (PKCδ) plays a crucial role in its CAR-dependent adhesion dynamics, leading to enhanced E-cadherin endocytosis (10). Moreover, CAR associates with β integrins, and this requires the CAR cytoplasmic domain (14). CAR has also been implicated in regulation of cell-extracellular matrix (ECM) adhesion as increased activation of β1 and β3 integrins was shown to be a direct consequence of CAR-induced p44/42 activation (14). CAR interactions with the actin cytoskeleton suggest that it might be involved in additional processes such as cell migration. Indeed, CAR binds actin and microtubules and it interacts with the F-actin regulatory kinase Rho-associated Protein Kinase (ROCK) (15–17).

In addition to regulating cell-cell homeostasis, CAR is emerging as a key player in disease states such as inflammation and cancer (8). Phosphorylation of the cytoplasmic tail of CAR promotes trans-epithelial migration of leukocytes in inflammation (18). CAR levels are altered at both early and late tumor stages and variability in its role in tumor progression may depend on pre-existing endogenous CAR levels (19, 20). Like other JAMs, CAR has a dual role as it presents both a tumor suppressive and tumor promoting role according to the type of cancer (17, 21). CAR expression is upregulated in lung squamous cell carcinoma and adenocarcinomas and CAR expression level is correlated with poorer prognosis (22, 23). This makes CAR an attractive target for cancer treatment. In line with this, inhibition of CAR expression in lung cancer cells decreases tumor volume in Severe Combined Immuno Deficiency (SCID) mice and high CAR expression promotes expression of mesenchymal markers, suggesting it could play a role in EMT (20). CAR depletion in human lung cancer cells results in reduced anchorage-independent growth and tumor growth in mice (24). CAR not only contributes to tumor development, but also to treatment resistance as it has been identified as a marker of cancer stem cells in non-small lung cancer (25).

In this study we aimed to define whether changes to CAR levels as observed in lung cancer, could contribute to the metastatic potential *via* regulation of cell-cell and cell-ECM adhesion. Our data demonstrate that CAR overexpression promotes cell adhesion to ECM proteins and cell invasion into 3D collagen gels without affecting classical epithelial to mesenchymal transition (EMT) markers. Overexpression of WTCAR promotes tumor growth *in vivo* and cell adhesion in the lung after intravenous injection. Mechanistically, we show that phosphorylation of CAR is responsive to increasing extracellular stiffness. Overexpression of WTCAR results in increased β1 integrin activity through activation of the GTPase Ras-related Protein 1 (Rap1), leading to enhanced CAR-dependent adhesion. Immunoprecipitation experiments further show that CAR forms a complex with the focal adhesion proteins Src, Focal Adhesion Kinase (FAK) and paxillin and that this requires phosphorylation of CAR. Taken together, these data suggest that CAR contributes to lung cancer metastasis *via* promotion of cell-matrix adhesion.

## Methods

### Antibodies and Reagents

Anti-integrin β1 (12G10, Santa Cruz), anti-active β1 integrin (9EG7, Merck Millipore), anti β-catenin [Santa Cruz (IF) and BD Bioscience (WB)], anti-CAR antibody (Santa-Cruz), anti E-Cadherin (Abcam), anti-FAK (Cell signalling), anti-green fluorescent protein (GFP) (26), anti- Heat Shock Cognate 70kDa (HSC70) (Sigma-Aldrich), anti-paxillin (BD Bioscience), anti phospho-FAK (Y397, Cell signalling), anti-phospho-paxillin (Y118, Cell signalling), anti-phospho-src (Y418, Millipore), anti-rap1 A/B (R&D Systems) and anti-src (Millipore) antibodies were used for western blot. Adenovirus Type5 fiberknob (Ad5FK) was produced and purified as previously described (27). CAR thr290/ser293 polyclonal antibody was developed by Perbioscience (Thermofisher) using the peptide Ac-RTS (28)AR(pS)YIGSNH-C and was affinity purified before use. DAPI (Sigma Aldrich) was used as nuclear stain for immunofluorescence. Anti-mouse-HRP, anti-rabbit-HRP, anti-goat-HRP were from DAKO, anti-mouse-568, anti-rabbit-568, anti-goat-568 and phalloidin-647 were all obtained from Invitrogen. Inhibitors to FAK (PF228), Rap1A (GGTI298) and src (PP2) were all obtained from Tocris.

### Plasmids and Primers

CAR phospho-mutants (T290A & S293A, non-phosphorylated (AACAR) and T290D & S293D, phospho-mimetic (DDCAR) in both GFP lentiviral backbones and Glutathione S-transferase (GST) expression vectors were described previously (10). RalGDS-RBD-GST construct was kindly gifted by Dr Ritu Garg (King’s College London). Luciferase-mStrawberry lentiviral plasmid and pMDL, RSV-Rev and CMV-VSVG packaging plasmids were kindly gifted by Dr Scott Lyons (Cold Spring Harbor Laboratories). The *CXADR* CRISPR Guide RNA targeting sequence TAGATACGCAGTTTCCCCTT cloned into pSpCas9 BB-2A-GFP (PX458) lentiviral construct and acquired from Genscript. The following primers were used for qPCR: CAR primer forward 5’ AAGTGACGCGAGTTCACCTG 3’CAR primer reverse 5’AGATGTTCAAGACCTGTACACTG 3’ 18S primer forward 5’-CCCATCACCATCTTCCAGGAGC -3’ 18S primer reverse 5’-CCAGTGAGCTTCCCGTTCAGC -3’.

### Cell Culture

LLC1 murine Lewis Lung Carcinoma epithelial cells (29), CMT-167 murine carcinoma alveogenic epithelial cells (30) were a kind gift from Prof K.Hodivala-Dilke (Barts Cancer Institute, QMUL, London) and Human Embryonic Kidney 293T (HEK293T) cells (purchased from ATCC) were cultured in high glucose DMEM containing 10% FCS, supplemented with 2mM glutamine. HEK293T packaging cells were used to generate lentiviral particles for CAR and GFP lentiviral expression using GFP, WTCAR-GFP and AACAR-GFP constructs. CAR-CRISPR cell lines were established *via* transient transfection of LLC and CMT parental cells with *CXADR* CRISPR Guide RNA vector carrying a GFP tag. The GFP-tagged cells were sorted 24 h post-transfection using flow cytometry to obtain a homogenous but non-clonal cell population. CMT and LLC cells were treated with Ad5 FK (100μg/mL for 2.5h), FAK inhibitor (PF228; 1μM for 4h), Rap1A inhibitor (GGTI298, 10μM for 30min) or Src inhibitor (PP2, 5μM for 4h). PP2 also inhibits Lck and Fyn members of the Src family of tyrosine kinases (31) but PP2 has been previously shown to effectively inhibit Src activity (32) at concentrations used here (33). Effective inhibition of Src was proven by reduced pY418Src. Ad5FK was used at concentrations previously shown to block CAR homodimerisation (18). GGTI298 has been effectively used to inhibit Rap1 processing (34, 35).

### Generation of Lentiviral Virus From HEK-293T Cells

HEK-293T cells were plated at 40% confluency 24 hours prior to transfection. A transfection mixture containing a total of 7.5 μg DNA (2.1 µg pCMV8.91, 0.7 µg pMD.G and 3.75 µg of various lentiviral constructs) was mixed in 500 µL of OptiMEM. Subsequently, 22.5 µL of PEI transfection reagent was added in the transfection mixture (3:1 ratio to total DNA). The DNA-PEI mix was vortexed and incubated for 15 minutes at room temperature before being added to HEK-293T cells with OptiMEM. After incubation with the transfection mixture for 5 hours at 37°C, OptiMEM was replaced with complete media. Lentivirus was harvested after 48 hours by removing the media and centrifugation at 1200 rpm for 3 minutes to remove any HEK293T cells. Viruses were then filtered through 0.4 µm sterile filters and stored in 1 mL aliquots at -80°C.

### Lentiviral Infection to Generate Stable Cell Lines

CMT and LLC cells were plated to 40% confluency 24 hours prior to viral infection. Polybrene (8 mg/mL) was added into normal growth media to increase the efficiency of viral infection. 1-4 mL of lentivirus were added to the cells and left to incubate at 37°C for 24-72 hours. Media was replaced 24-48 hours post-infection to remove the virus and cells were grown and passaged.

### GFP-Trap Immunoprecipitation

LLC and CMT cells expressing GFP-tagged proteins were lysed in IP lysis buffer (pH 7.4, 50 mM Tris, 150 mM NaCl, 50mM NaF, 1 mM EDTA, 1% Triton X-100, 1% NP40, PI cocktail). Lysates were incubated with 1:1 of GFP-trap^®^beads (Chromotek) and agarose resin on a rotator at 4°C for 2 hours. Beads were washed with IP lysis buffer and immunocomplexes were separated using SDS-PAGE and immunoblotted for either β1 integrin, FAK, paxillin, Src or GFP.

### GST Pulldown Assay

GST-fusion constructs were expressed in *E. coli* BL21 competent cells using conditions recommended by the manufacturer (Amersham Pharmacia Biotech). Pulldown assays were carried out using WTCAR-GST, AACAR-GST and DDCAR-GST cytoplasmic tail constructs as previously described (10). LLC and CMT cells were cultured in 10 cm dishes until 100% confluent and lysed in 500 µl IP buffer containing protease and phosphatase inhibitor cocktails (1:100). Cell lysate proteins were collected as supernatant after centrifuging at 13,000 rpm for 10 minutes at 4°C. 50 μl of each lysate was kept aside for use in loading controls while the rest were incubated with pre-washed GST or CAR-GST beads for 3 hours at 4°C on a rotator. Following incubation, the unbound fractions were removed and the beads washed three times with IP buffer before boiling for 5 minutes in 50 μl of 2X SDS sample loading buffer containing β-mercaptoethanol (1:50). 40 μl of samples were loaded onto 10% polyacrylamide gels and immunoblotted.

### Western Blotting

CMT and LLC cells were lysed in RIPA buffer (pH 7.4, 10 mM Tris Base, 150 mM NaCl, 1mM EDTA, 1% Triton X- 100) containing β-mercaptoethanol (1:100). Lysates were subjected to SDS-PAGE and blotted using PVDF membrane. Blots were blocked and probed using 5% skimmed milk powder or Bovine Serum Albumin in TBS-0.1% Tween. Proteins were detected by ECL chemiluminescence kit (BioRad) and directly imaged using the BioRad imager digital imaging system.

### Immunofluorescence

CMT and LLC cells were plated on 13mm coverslips and incubated overnight in normal growth media. Cells were washed 1x with PBS and then fixed using 4%PFA in PBS for 10 minutes at room temperature and then washed three times with PBS. Cells were then stored or permeabilised using 0.25% TritonX-100 in PBS for 5 minutes and washed again three times with PBS. Following permeabilization, coverslips were blocked with 5% BSA/PBST for 1 hour at room temperature. Incubation of cells with primary antibody diluted in 5% BSA/PBST was then carried out in a dark humid chamber placed at 4°C overnight. Cells were incubated with secondary antibodies and DAPI diluted in 5% BSA/PBST for 1h at room temperature. Coverslips were mounted on slides using FluorSafe mounting media (Calbiochem).

### Confocal Microscopy

The slides were analysed using a A1R laser scanning confocal microscope (Nikon) with a 60x/1.4Plan-APOCHROMAT oil immersion objective. Images were acquired in ND2 format, exported as TIFFs, and analysed in Image J. Confocal microscopy Images of fixed cells were acquired on a Nikon A1R inverted confocal microscope (Nikon Instruments UK) with an environmental chamber maintained at 37°C/5% CO2. Images were taken using a 40x or 60x Plan Fluor oil immersion objective (numerical aperture of 1 and 1.4, respectively). Excitation wavelengths of 488 nm (diode laser), 561 nm (diode laser) or 640 nm (diode laser) were used. In experiments where multiple cell lines were analysed from the same cell type, all images were acquired at identical laser settings to permit comparison of intensities. Images were acquired using NIS-Elements imaging software (v4) and were saved in Nikon Elements in the.ND2 format. Image processing was carried out in Fiji processing software (36).

### Fluorescence Recovery Activated Photo Bleaching (FRAP)

CMT cells expressing WTCAR-GFP and AACAR-GFP were used for FRAP experiments. CMT cells expressing WTCAR-GFP were treated with DMSO/anti-src (PP2, for 4h) and used for FRAP experiments in presence of anti-Src/DMSO. Regions of interest (23) were drawn across CAR-positive cell-cell junctions and photobleached by a bleach pulse (0.5 second) at 80% laser intensity at 488 nm. Recovery of fluorescence within the ROI was monitored over 6 min. Background and reference ROIs were selected for background and reference correction. 10 cell-cell junctions were averaged to generate one FRAP curve for a single experiment. Fluorescence recovery of CAR-GFP was analysed using the NIS-Elements Advanced Research software. The experimental data were fitted using the one-phase decay in Graphpad Prism.

### Cell Matrix Adhesion Assay

CMT and LLC cells were plated on Collagen (Rat Tail Type I) or Matrigel and allowed to adhere for 2h at 37°C. Cells were fixed with 4%PFA and nuclei were stained *via* incubation with DAPI (1:1000) for 10min. Fluorescent images were acquired on Evos FL Auto 2 fluorescent microscope (Invitrogen). Tile-scans were obtained using a 4x air objective with 3.2 MP CMOS camera. Excitation using DAPI LED light cube was used. Images were acquired using EVOS software (v2). Tiles were knitted into.TIFF files using FIJI software and total cell count was obtained by thresholding for nuclear stain followed by automated counting. 35mm low stiffness μ-dishes (1.5kPa and 28 kPa) were obtained from Ibidi.

### Cell Proliferation Assay

LLC and CMT cells were fixed with 4% PFA/PBS for 10 min at 4h, 24h and 48h post-plating. Nuclei were stained with DAPI to enable cell quantification. Images were acquired as described for cell matrix adhesion assay.

### Inverted Invasion Assay

Transwell inserts with 8 μm wide pores were filled with a gel comprised of Collagen (Rat Tail Type I 1.6 mg/mL), Fibronectin (10μg/mL) and FCS (2%). LLC and CMT cells plated on top of each transwell were left to invade through the gel for 72h following a FCS concentration gradient. Transwells were fixed with 4%PFA and nuclei stained with DAPI. Confocal sections were taken every 1.5 μm in 5 independent fields per transwell with a 20x dry objective in a Nikon Eclipse Ti-E inverted microscope with A1R Si Confocal system using Nikon Software Elements. To quantify invasion levels, images acquired in the DAPI channel were imported on Fiji software. Cell count was obtained by thresholding for nuclear stain followed by automated counting. The percentage of total invading cells present at each depth in the collagen gel was determined. This was done by dividing the number of cells present in each layer by the sum of invading cells present in all layers.

### Spheroid Assays

CMT cells were re-suspended in DMEM supplemented with 0.5% FCS and methylcellulose (37) to generate spheroids *via* the hanging drop method. Spheroids were left to form for 48 h at 37°C. A collagen mix containing 2 mg/ml collagen (Rat Tail Type I collagen), 20 mM Hepes, 10 mM fibronectin, 17.5 mM NaOH and OptiMEM was made to embed the spheroids. Phase-contrast images were acquired using the Evos FL Auto 2 fluorescent microscope with a 10x objective at 0, 24 and 48 hours. Cell invasion was quantified using Fiji software. The acquired images (.TIFF format) were imported in Fiji, a region of interest was manually drawn around each spheroid and the area quantified. 0h post-embedding spheroids were only comprised of a spheroid core, whereas 24 and 48 h post-embedding cells started leaving the core and spheroids were comprised of invading cells in addition to a spheroid core. Regions of interest were drawn around the entire spheroid (including invading cells). Spheroid cell invasion was quantified by dividing the spheroid area at 24h or 48 h by the spheroid area measured at 0h post-embedding.

### *In-Vivo* Experiments

The use of animals for this study was approved by the Ethical Review Committee at King’s College London and the Home Office, UK. All animals were housed in the Biological Support Unit (BSU) located in New Hunt’s House at King’s College London. All experiments were carried out under project license no. P9672569A and personal license no. I83A1F143. For subcutaneous tumors, 7.5x10^6^ CMT or LLC cells were re-suspended in 200 μl PBS. Cells were injected subcutaneously into the shaved flank of immunocompetent C57BL/6 mice (male, 7-8 weeks old, 20 g weight). Tumors were allowed to grow until they reached a maximum diameter of 20 mm. A Vernier caliper was used to measure perpendicular tumor diameter every three days and tumor volumes were calculated using the following formula: V = (W(2) × L)/2. For experimental metastasis models, 7.5x10^5^ CMT or LLC cells expressing a luciferase mStrawberry-tagged construct were re-suspended in 150 μl of PBS and injected into the mouse tail vein of immunocompetent C57BL/6 mice (male, 7-8 weeks old, 20 g weight). To detect luminescent cells, each mouse was injected intra-peritoneally with 200 μl of D-luciferin (PerkinElmer, 0.15 mg Luciferin/g body weight) PBS solution before *in vivo* imaging. Mice were imaged at 4h and 24h post-injection using the IVIS spectrum imaging system (PerkinElmer) (38). A region of interest was drawn around each luminescent signal present in the lung and quantified using the Living Image^®^ software and measured in Total Flux [= radiance (photons/sec) in each pixel summed over the ROI area (cm^2^) x 4π].

### Tissue Processing

Tumors extracted from mice were fixed with 4%PFA prior to paraffin wax embedding. 10 μm thick sections were obtained and used for DAB staining. Paraffin-embedded sections were dewaxed and antigen retrieval was carried out *via* 20min incubation with sodium citrate buffer (0.0874 M sodium citrate, 0.0126 M citric acid, pH 6) in a pressure cooker at 95°C. Endogenous peroxidase activity was blocked *via* 10 *min* incubation in hydrogen peroxide (3% in TBS). Non-specific binding was blocked *via* incubation with TBS-1%BSA-1%FBS blocking solution. Tissues were incubated with primary antibody at 4°C overnight. Secondary HRP-conjugated antibody was added for 1h at room temperature. DAB staining was visualised by adding DAB developing solution for up to 20 min (Dako, Liquid DAB+Substrate Chromogen system). Tissues were counterstained using haematoxylin for 1 sec. Tissues were dehydrated and mounted with DPX mounting medium (Sigma-Aldrich).

### Statistical Analysis

Data values are expressed as mean ± standard error of mean (s.e.m). All statistical tests were performed using GraphPad Prism, version 8. Student’s t-test was used for comparing two groups for statistical analysis. One or two-way analysis of variance (ANOVA) with *post hoc* test was used for multiple comparisons. Statistically significant values were taken as * = p < 0.05, ** = p < 0.01, *** = p < 0.001, **** = p < 0.0001 and were assigned in specific figures and experiments as shown.

## Results

### CAR Regulates Lung Cancer Cell Invasion But Does Not Alter EMT Phenotypes

**Figure 1:**
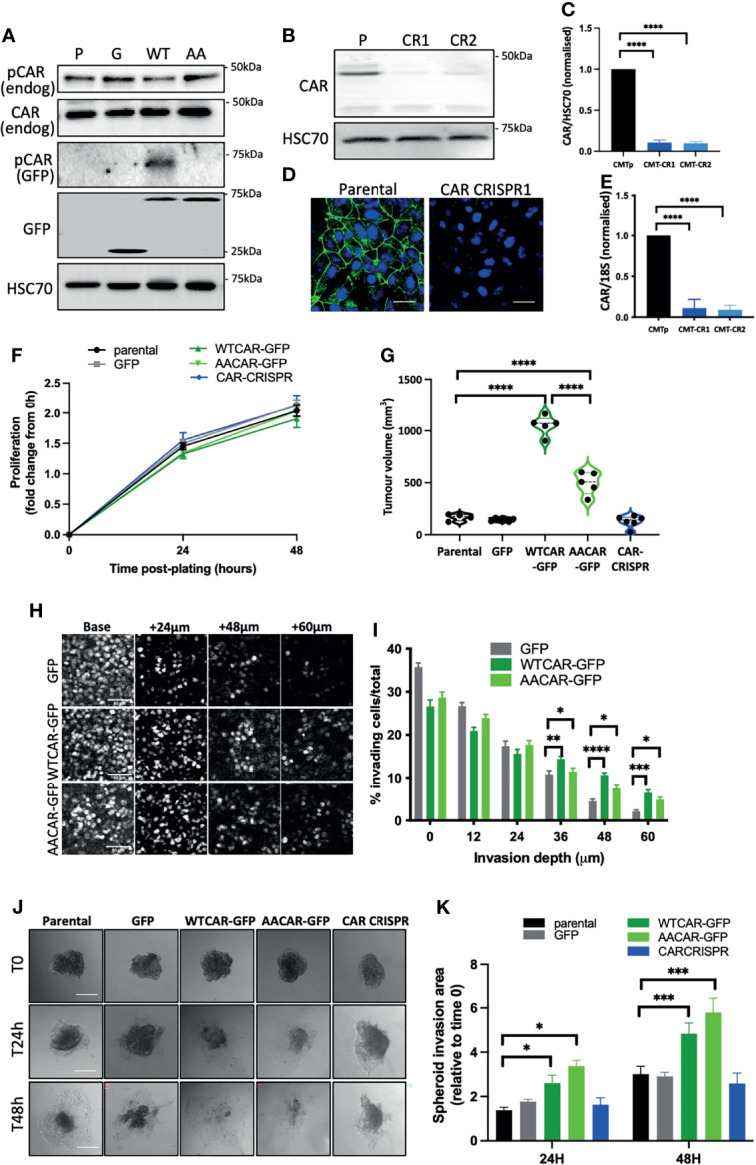
CAR promotes lung cancer progression. **(A)** Representative Western blots of specified proteins in CMT 167 cells, parental (P), GFP expressing **(G)**, WTCAR-GFP (WT) and AACAR-GFP (36). **(B)** Representative Western blots of CAR levels in parental (P) and 2 different CAR CRISPR CMT 167 cell populations (CR1, CR2). **(C)** Graph shows quantification of CAR levels as in **(B)** from 4 independent experiments. **(D)** Representative confocal images of parental and CAR CRISPR cells stained for CAR (green) and DAPI (blue). Scale bars are 10 μm. **(E)** CAR transcript levels in parental and CRISPR cells by qPCR relative to 18S control. Pooled data from 3 independent experiments. **(F)** Proliferation in specified CMT 167 cells 24h and 48h post-plating. Data is representative of 3 independent experiments and from 4 replicates per cell line per time point. **(G)** Tumor volume in mice subcutaneously injected with specified CMT 167 cells. Violin plots shown with each point represents one animal (n=5 per group). **(H)** Representative confocal images of nuclei stained in specified CMT 167 cell lines at intervals through 3D inverted invasion assay. Scale bars are 50 μm. **(I)** Quantification of invasion data from **(H)** at different depth intervals, pooled from 4 different wells per condition, representative of 3 independent experiments. **(J)** Representative phase contrast widefield images of specified CMT 167 cell spheroids images over time. Scale bars are 100 μm. **(K)** Quantification of spheroid area over time relative to time 0. 10 spheroids per cell line analysed; representative of 3 independent experiments. All values are mean ± SEM. P values *p < 0.05,**p < 0.01, ***p < 0.0005, ****p < 0.0001.

To explore whether CAR plays a role in tumorigenesis and EMT we generated a panel of epithelial-like CMT 167 mouse lung adenocarcinoma cells overexpressing WT or AACAR (**Figure 1A**) and where CAR is removed using CRISPR (**Figures 1B–E**). As a cell-cell adhesion molecule, CAR has previously been suggested to play a role in EMT. Notably we did not observe any changes in cell-cell adhesion behaviour under normal growth conditions. However, to more formally address whether changes to EMT may be occurring, we assessed expression of key characterised EMT markers E-Cadherin, β-catenin and vimentin in these cells. Confocal images demonstrated colocalization of both WTCAR and AACAR with E-Cadherin at cell-cell adhesion sites (**Supplementary Figure 1A**). Overexpression or deletion of CAR did not change levels or localisation of E-cadherin or β-catenin as quantified from confocal images and western blots (**Supplementary Figures 1A–C**). Moreover, vimentin was detected in CMT 167 cells, despite their epithelial-like appearance, but the expression of this protein was unaltered following manipulation of CAR levels (**Supplementary Figure 1D**), further indicating that CAR does not contribute to EMT changes. Whilst our data from fixed cells suggested that WTCAR and AACAR were similarly distributed at cell-cell adhesions, we postulated, based on our previous data in normal lung epithelial cells, that dynamics of CAR itself would be altered upon phosphorylation of the cytoplasmic tail (10). Analysis of CAR dynamics at the plasma membrane using fluorescence recovery after photobleaching (FRAP) revealed significantly slower recovery of AACAR compared to WTCAR at cell-cell adhesions (**Supplementary Figures 1E, F**) indicating phosphorylation promotes CAR movement at the membrane but that this does not result in a tangible change in cell-cell adhesion properties. Functional analysis of this panel of cells showed a doubling time of approximately 48 hours in parental cells that did not significantly change in CAR overexpressing or CRISPR cells over this time (**Figure 1F**). However, subcutaneous injection of cells into immunocompetent mice revealed a significant increase in tumor growth *in vivo* in WTCAR and AACAR cells, with WTCAR cells showing the highest proliferative potential (**Figure 1G**). This data demonstrates that higher levels of CAR promote tumor growth, and this may be specific to 3D microenvironments. To determine whether this enhanced proliferative capacity correlated with invasive potential, we analysed invasion as measured using 3D inverted invasion assays and 3D spheroids. In both cases, WTCAR and AACAR promoted invasion in CMT cells compared to controls (**Figures 1H–K**). Collectively these data demonstrate that enhancing CAR expression can drive a pro-tumorigenic phenotype within 3D environments.

### CAR Expression Promotes Cell-Matrix Adhesion

**Figure 2:**
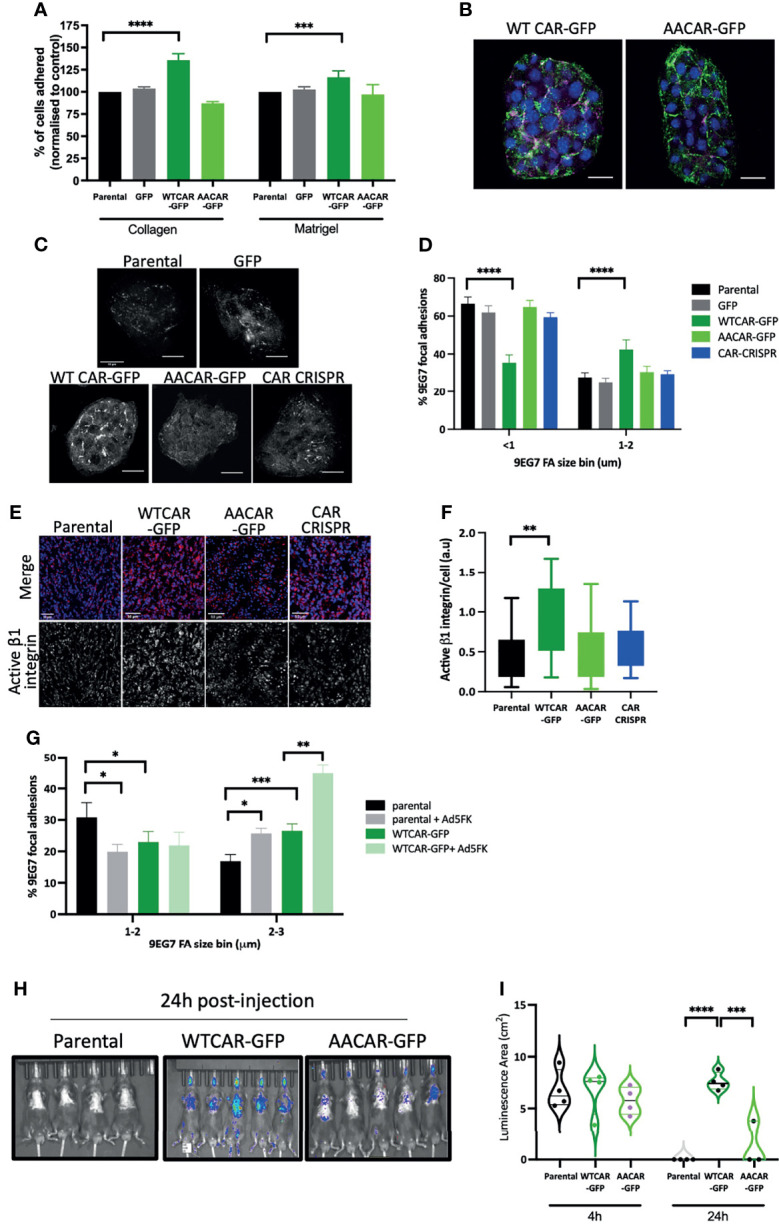
CAR promotes cell-matrix adhesion. **(A)** Graphs showing % adhesion to collagen and Matrigel of indicated cell lines 2h post-plating. Data is from 3 wells per cell line, representative of 3 independent experiments. **(B)** Representative confocal images of WTCAR-GFP and AACAR-GFP CMT cells (CAR; green) stained for active β1 integrins (9EG7; magenta). Scale bars are 20 μm. **(C)** Representative confocal images of indicated cell lines stained for active β1 integrins (9EG7). Scale bars are 50 μm. **(D)** Quantification of images as in **(C)** from at least 10 different fields of view. Data is presented as % of active integrin adhesions categorised in sizes. Representative of 3 independent experiments. **(E)** Representative confocal images of tissues from subcutaneous tumors of indicated cell lines, stained for active integrins (9EG7, red) and DAPI (blue). Scale bars are 50 μm. **(F)** Quantification of images as shown in (D or E)?. Data is from 6 tumors per cell line, and 4 fields of view per tumor. **(G)** Quantification of 9EG7 staining as in (B,C what about 9eg7 fk images)? from parental or WTCAR-GFP cells with or without Ad5FK pre-incubation. Data is from at least 10 different fields of view per condition. Data is presented as % of active integrin adhesions categorised in sizes. Representative of 3 independent experiments. **(H)** Images of indicated cell lines expressing luciferase 24 hours after intravenous injection into animals. Representative of 2 independent experiments. **(I)** Quantification of data as in **(H)** Violin plots shown with each point represents one animal (n=4-5 per group). All values are mean ± SEM. P values *p < 0.05,**p < 0.01, ***p < 0.0005, ****p < 0.0001.

As our data demonstrated no change in cell-cell adhesion but enhanced invasion when CAR is overexpressed, we next explored whether CAR may be mediating a pro-invasive phenotype through contributions to cell-matrix adhesion. We firstly explored whether initial attachment to ECM proteins was altered across the CMT cell panel with a particular focus on those ECM proteins within the stroma (collagen I) or surrounding the solid tumor (basement membrane). WTCAR overexpression promoted initial CMT cell attachment to both Collagen I and Matrigel as measured 2 hours post-plating (**Figure 2A**). To explore whether this phenotype was associated with higher levels of active β1 integrins, that are the major receptor family for these ECM proteins, cells were fixed and stained with an antibody that recognizes the active conformation of these receptors (9EG7). Images revealed that neither WTCAR-GFP nor AACAR-GFP localised to active integrin containing adhesions (**Figure 2B**). However, quantification of data revealed that WTCAR but not AACAR expressing cells showed a significant enhancement in larger activeβ1 integrin containing focal adhesions (**Figures 2C, D**). In agreement with this, we also observed significantly increased activeβ1 integrins in solid tumors following subcutaneous injection of WTCAR overexpressing cells (**Figures 2E, F**). To test whether CAR engagement *in trans* at cell-cell adhesions contributed to this integrin-based adhesion phenotype, we treated cells with recombinant Ad5FK to block CAR *in trans* binding as we have previously (18). Ad5FK enhanced active β1 integrins in both parental and WTCAR expressing CMT cells (**Figure 2G**), suggesting CAR-CAR homodimers need to be disengaged at cell-cell adhesions for CAR to promote cell-matrix adhesion. To determine if this enhanced adhesion phenotype was also seen *in vivo*, we performed IV injections of CMT cells expressing a luciferase reporter and analysed retention of cells in the lung over 24 hours. Significantly enhanced retention of WTCAR cells in lung was seen after 24 hours (**Figures 2H, I**) suggesting enhanced cell-matrix stability leads to increased metastatic potential.

### CAR Expression Promotes Adhesion and Invasion in Mesenchymal Cells

**Figure 3:**
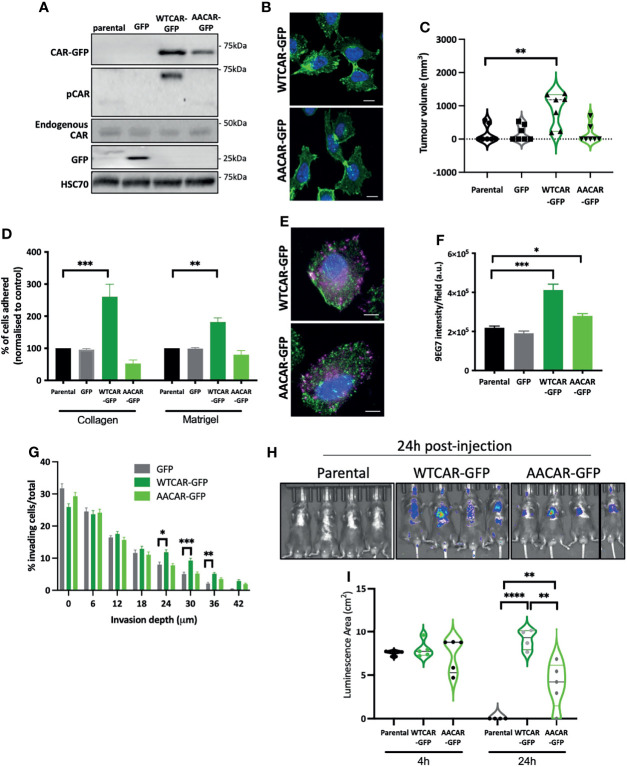
CAR promotes adhesion and invasion in mesenchymal cells. **(A)** Representative Western blots of specified proteins in LLC cells, parental, GFP expressing, WTCAR-GFP and AACAR-GFP. **(B)** Representative confocal images of WTCAR-GFP and AACAR-GFP expressing LLC cells. CAR is shown in green and DAPI in blue. Scale bars are 10 μm. **(C)** Tumor volume in mice subcutaneously injected with specified CMT 167 cells. Violin plots shown with each point represents one animal (n=7-8 per group). **(D)** Graphs showing % adhesion to collagen and Matrigel of indicated cell lines 2h post-plating. Data is from 3 wells per cell line, representative of 3 independent experiments. **(E)** Representative confocal images of WTCAR-GFP and AACAR-GFP LLC cells (CAR; green) stained for active β1 integrins (9EG7; magenta). Scale bars are 10 μm. **(F)** Quantification of images of specified cell lines stained for active β1 integrins (9EG7). Data is from at least 10 fields of view (5-10 cells per field) per condition. Representative of 3 independent experiments. **(G)** Quantification of inverted invasion data from specified cell lines at different depth intervals, pooled from 4 different wells per condition, representative of 3 independent experiments. **(H)** Images of indicated cell lines expressing luciferase 24 hours after intravenous injection into animals. Representative of 2 independent experiments. **(I)** Quantification of data as in **(H)**. Violin plots shown with each point represents one animal (n=4-5 per group). All values are mean ± SEM. P values *p < 0.05, **p < 0.01, ***p < 0.0005, ****p < 0.0001.

Our data show that CAR engagement at junctions negatively regulates the ability of epithelial-like carcinoma cells to activate integrins and undergo invasion. We therefore hypothesised that overexpressing CAR in mesenchymal lung cancer cells would promote adhesion and invasion. To test this, we generated LLC mouse lung carcinoma cells overexpressing WT and AACAR (**Figure 3A**), noting these cells express significantly reduced endogenous CAR compared to CMT 167 cells. Both WTCAR and AACAR localised to the membrane and was enriched at sites of transient cell-cell contact (**Figure 3B**). Analysis following subcutaneous injection of these cells revealed enhanced tumor growth in WTCAR overexpressing cells (**Figure 3C**), similar to that seen in CMT cells (**Figure 1G**). WTCAR overexpression also enhanced initial LLC cell attachment to Collagen I and Matrigel (**Figure 3D**). To determine whether CAR was associated with integrin-containing focal adhesions in mesenchymal lung cancer cells, WTCAR-GFP and AACAR-GFP overexpressing cells were stained with antibody to active β1 integrins. Images demonstrated no clear colocalisation of CAR and β1 integrins in either cell line (**Figure 3E**). However, significantly enhanced β1 integrin activation was measured in WTCAR-GFP overexpressing cells with a smaller increase seen in AACAR cells (**Figure 3F**) as also seen in CMT cells. WT and AACAR overexpression also enhanced LLC cell invasion in 3D inverted invasion assays (**Figure 3G**). Finally, WTCAR overexpression increased LLC cell retention in the lung following IV injection as measured using luciferase reporters (**Figures 3H, I**). These data suggest that overexpression of CAR in mesenchymal cancer cells enhances cell-matrix adhesion and invasion, suggesting CAR-dependent effects on these phenotypes do not require CAR localisation to adherens and tight junctions.

### CAR Is Expressed Highly at Tumor Boundaries and Is Mechano-Responsive

**Figure 4:**
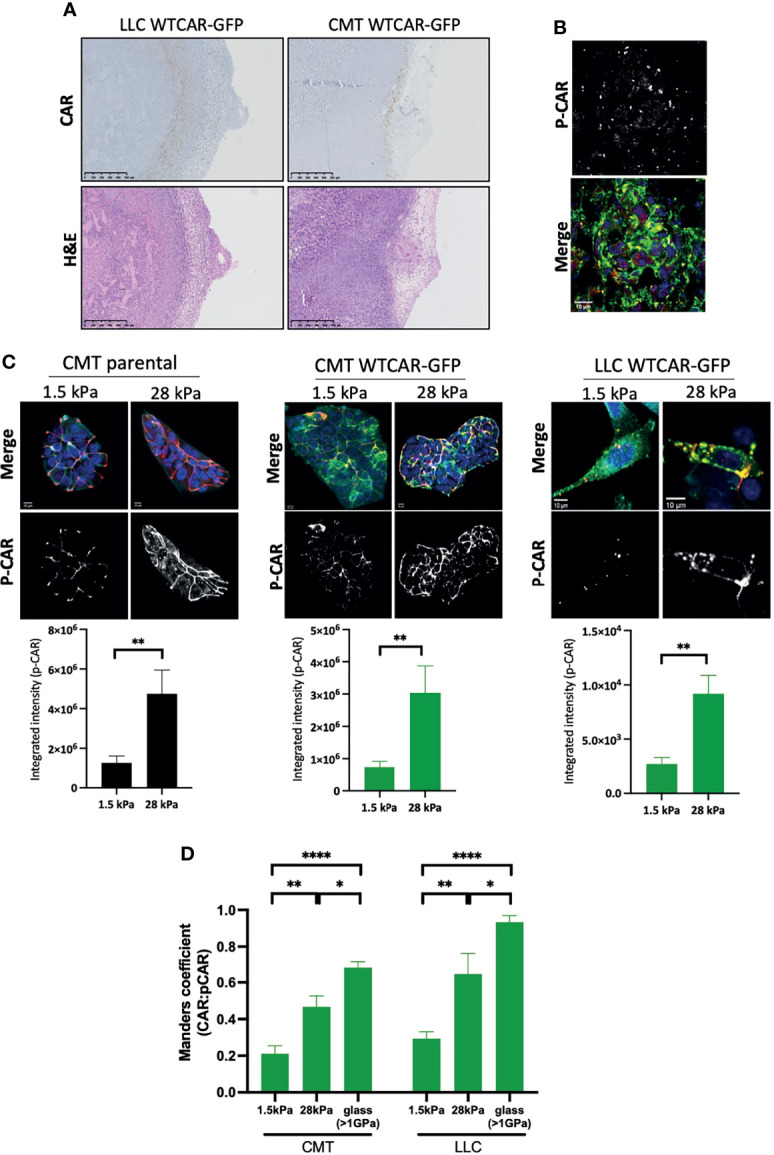
CAR is a mechano-responsive protein. **(A)** Representative images of sections from tumors formed following subcutaneous injection of LLC or CMT cells stably expressing WTCAR-GFP, stained for CAR (top panels, brown) or H&E (bottom panels). **(B)** Representative images of P-CAR staining of frozen sections of tumors formed following subcutaneous injection of CMT WTCAR-GFP expressing cell. Top panel shows P-CAR alone, bottom panel P-CAR (39), CAR-GFP (green) and DAPI (blue). **(C)** Representative confocal images of indicated cell lines plated on 1.5 kPa or 28 kPa stiffness plates, stained for P-CAR (bottom panels) and DAPI (blue). CAR is shown in green. Graphs beneath each image set show quantification of P-CAR from at least 10 fields of view per cell line, representative of 3 independent experiments. **(D)** Quantification of colocalization between CAR and P-CAR from images as in **(C)** from CMT and LLC cells expressing WTCAR-GFP. All values are mean ± SEM. P values *p < 0.05, **p < 0.01, ****p < 0.0001.

Our data demonstrate that high expression of CAR positively regulates tumor cell-matrix adhesion, and this may be distinct from the known role of CAR at cell-cell adhesion sites. To determine whether CAR expression levels correlated with sites of cell-matrix adhesion in tumors, we stained for CAR in tissue sections from subcutaneous CMT and LLC cell tumors from immunocompetent mice. An increase in CAR levels was observed at the edge of solid tumors where cells contact the ECM in both cell types (**Figure 4A**). Moreover, phosphorylated CAR was also readily detected at the boundary of these tissues (**Figure 4B**). In addition to the biochemical properties of the ECM, the stiffness of the matrix surrounding the tumor is increasingly recognized as a key factor contributing to tumor growth and invasive potential (40). Enhanced stiffness in the tumor microenvironment is sensed through integrin-based focal adhesions and has been associated with increased invasion in several tumor types. Given our data demonstrating that CAR contributes to cell-matrix interactions in a phosphorylation-dependent manner, we next determined whether CAR phosphorylation was responsive to changes in the matrix properties. To define this, we analysed CAR phosphorylation in cells plated on substrates of differing biomechanical properties; 1.5kPa (normal lung tissue stiffness), 28kPa (stiffness detected in several lung cancers) or glass (>1Gpa, standard non-physiological substrate). We analysed levels of pCAR as a function of CAR expression on a cell-by-cell basis to control for any variation in CAR expression levels. Data revealed significantly enhanced levels of CAR phosphorylation in cells on stiffer ECM (**Figures 4C**) and this correlated with higher CAR:P-CAR colocalization co-efficient in both CMT and LLC cells (**Figure 4D**). These data show that properties of the surrounding ECM can lead to altered levels and localisation of phosphorylated CAR, potentially promoting CAR-dependent cell-matrix adhesion and invasion.

### CAR Forms a Complex With Focal Adhesion Proteins and Promotes Rap1 Activity

**Figure 5:**
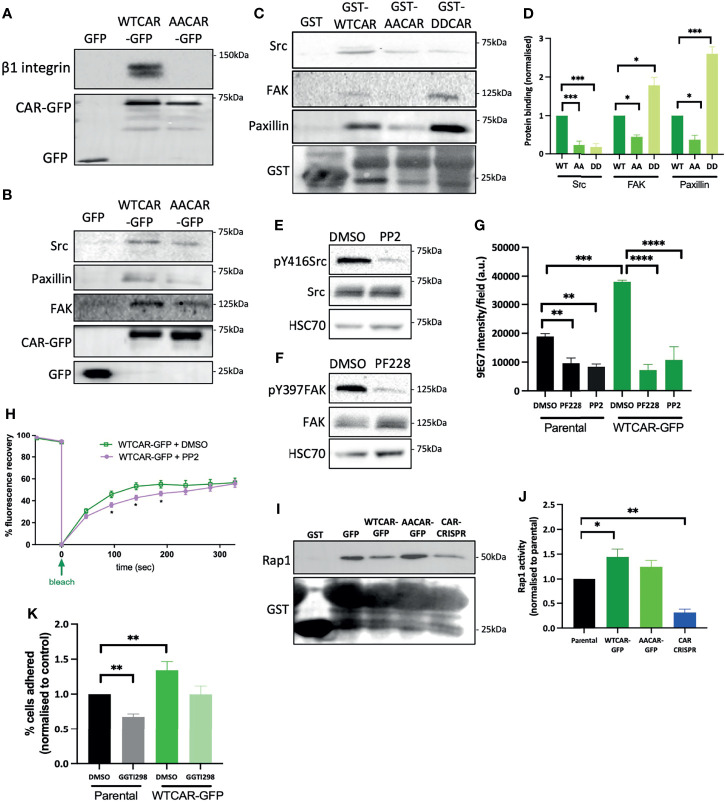
CAR forms a complex with focal adhesion proteins. **(A)** Representative western blot of immunoprecipitation of β1 integrins from specified cell lines probed for β1 integrin and GFP. Representative of 5 independent experiments. **(B)** Western blots of immunoprecipitation of GFP or GFP-CAR from lysates from specified cell lines probed for indicated focal adhesion proteins and GFP. Representative of 5 independent experiments. **(C)** Western blots of GST pulldowns using GST only or GST-CAR cytoplasmic tail (WT, AA, DD) using from lysates from CMT parental cells probed for indicated focal adhesion proteins and GST. **(D)** Quantification of blots as in C from 5 independent experiments. **(E, F)** Western blot of lysates from CMT parental cells treated with Src inhibitor [PP2, 1μM, **(E)**] or FAK inhibitor [PF228, 1μM, **(F)**] probed for indicated proteins. **(G)** Quantification of active β1 integrins (9EG7) from images of specified CMT cell lines treated with DMSO, PP2 or PF228. Data is from at least 10 fields of view (5-10 cells per field) per condition. Representative of 3 independent experiments. **(H)** Quantification of recovery curves of fluorescence intensity for FRAP data from WTCAR-GFP CMT cells +/-PP2. Data is from at least 15 different ROIs per cell line, shown as mean +/-SEM, representative of 3 independent experiments. **(I)** Representative Western blots of lysates from Rap1 activity pulldown assays from specified CMT cell lines, probed for Rap1 and GST. **(J)** Quantification of data as in **(I)**, pooled from 5 independent experiments. **(K)** Quantification of CMT parental and WTCAR-GFP cell adhesion to Matrigel after 2h, following pre-treatment with DMSO or Rap1 inhibitor (GGTI298, 5μM) normalised to control. Data is from 3 wells per cell line, representative of 3 independent experiments. All values are mean ± SEM. P values *p < 0.05, **p < 0.01, ***p < 0.0005, ****p < 0.0001.

In order to understand whether the observed CAR-dependent changes to cell-matrix adhesion were due to CAR physically engaging with focal adhesion proteins, we performed CAR immunoprecipitation experiments and probed for candidate proteins. We chose to focus on β1 integrins, Src, FAK and paxillin as these are core components of most focal adhesions formed in cells on collagen substrates and represent key regulatory elements to control of adhesion formation and dynamics. WTCAR, but not AACAR formed a complex with both β1 integrins (**Figure 5A**) and key focal adhesion components Src, FAK and paxillin (**Figure 5B**). Moreover, *in vitro* binding assays using the CAR cytoplasmic tail confirmed binding to FAK, Src and paxillin (**Figure 5C**) and further demonstrated dependence on phosphorylation of CAR at T290/S293 for these interactions as significantly lower binding was seen with AACAR vs WT or DD (phospho-mimic) CAR proteins (**Figure 5D**). To determine whether activity of these adhesion proteins may contribute to CAR-dependent cell-matrix adhesion, cells were treated with the Src inhibitor PP2 and the FAK inhibitor PF573228 (**Figures 5E, F**). Analysis of activeβ1 integrins revealed significantly reduced CAR-dependent β1 activation in the presence of both inhibitors (**Figure 5G**) indicating these proteins may be mediating CAR-dependent adhesion effects. To determine if Src activity might additionally play a role in controlling CAR mobility at the membrane, and thus its influence on cell-ECM adhesion, FRAP analysis was performed on WTCAR-GFP expressing cells in the presence of vehicle control or PP2. PP2 treatment resulted in significantly reduced CAR mobility at cell-cell adhesions (**Figure 5H**), supporting the notion that CAR-Src complex and Src activity may regulate the CAR-dependent effects on cell-ECM adhesion. Rap1 has also been previously shown to act downstream of the related family member JAM-A and controls integrin activation (41, 42). We therefore assessed Rap1 activity in the CMT 167 cell panel to understand if this GTPase may play a role downstream of CAR in driving integrin activity. WTCAR, but not AACAR overexpression led to significantly enhanced Rap1 activity as measured using pulldown assays, whereas depletion of CAR reduced Rap1 activity (**Figures 5I, J**). To further determine whether this enhanced activation of Rap1 contributed to CAR-dependent β1 integrin activation, cells were treated with the Rap1 inhibitor GGTI298 and adhesion to collagen I was tested as in previous experiments. Data revealed that treatment of cells with GGTI298 lead to suppression of CAR-induced adhesion (**Figure 5K**) indicating that enhanced integrin activation downstream of CAR is mediated by increased activity of Rap1. This data demonstrates that CAR acts to co-ordinate focal adhesion signalling through Src and Rap1.

## Discussion

**Figure 6:**
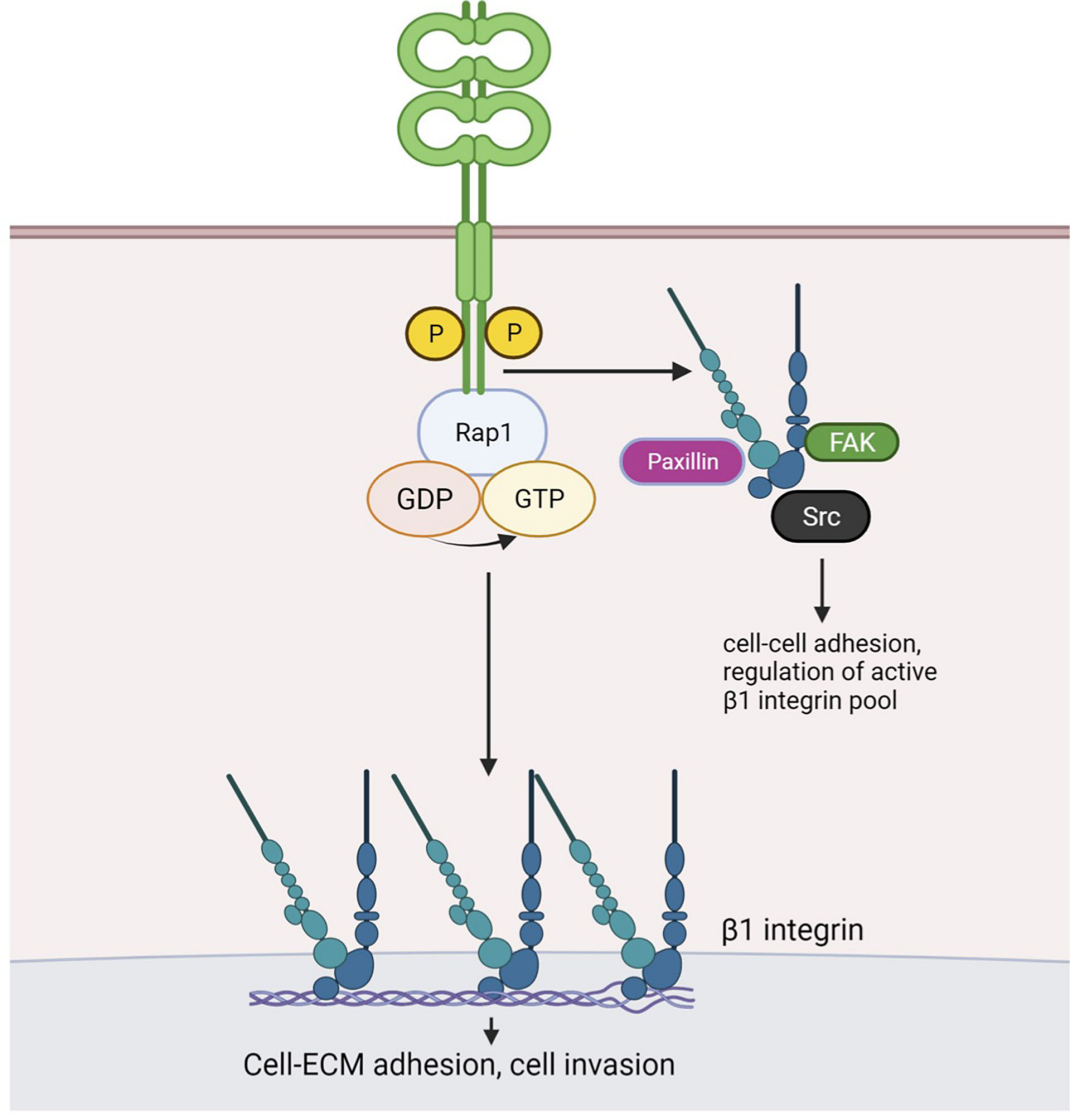
Working model for CAR-dependent regulation of lung cancer cell adhesion. Phosphorylated CAR promotes β1 integrin and Rap1 activation. Similarly to JAM-A, dimerised CAR may promote interaction of scaffold protein MAGI-1 and the Rap1 GEF PDZ-GEF2, resulting in Rap1 activation. CAR-mediated Rap1 activation promotes cell-ECM adhesion and may enhanceβ1 integrin activity. The latter may in turn be involved in CAR-mediated promotion of cell invasion. Phosphorylated CAR may recruit β1 integrins and/or focal adhesion components Src, FAK and Paxillin to the membrane to regulate cell-cell adhesion and to distally control focal adhesion activity. Figure created with BioRender.com.

Our study provides evidence that CAR contributes to several aspects of lung cancer progression including tumor growth, adhesion and invasion. Importantly, we show these effects of CAR overexpression do not require formation of adherens or tight junctions, where CAR has been previously studied. Multiple studies have investigated the role of CAR in tumor growth; however, these reports did not analyse whether CAR homodimerization *in trans* and factors dictated by the tumor microenvironment influenced this phenotype. We propose a model based on our data (**Figure 6**) whereby CAR activates the integrin-regulator Rap1 and associates with focal adhesion molecules and β1 integrins to influence cell-matrix adhesion leading to enhanced tumor invasion and metastasis. CAR may undergo dimerization *in cis* to bring scaffold molecule MAGI-1 and Rap GEF PDZGEF-2 together to activate Rap1. We speculate that phospho-CAR is more mobile on the membrane, and under these conditions can distally regulate integrin activity, without localizing to focal adhesions, *via* recruitment of focal adhesion molecules and/or β1 integrins to the membrane.

CAR expression levels have been shown to differ depending on the stage of cancer progression. For example, CAR up-regulation has been implied to promote carcinogenesis in early-stage breast cancer and breast cancer precursor cells (43, 44). However, CAR is downregulated in advanced disease stages displaying loss of differentiation in several tumor types (21, 45–48). Similarly, CAR levels are high during early carcinogenesis in colon adenomas and decreased during cancer dissemination in colon cancer metastases (49). However, changes in expression and function of CAR during carcinogenesis may be tumor-specific and both positive and negative expression contribute to different aspects of tumor progression (19). Our data demonstrate that CAR expression levels are highest at the boundary between the tumor edge and the stroma, in agreement with another recent study (28). Moreover, we show that CAR is a mechanosensitive protein and undergoes enhanced phosphorylation in response to stiffness. This coupled with our discovery that CAR can promote integrin activation and enhanced adhesion both *in vitro* and *in vivo* suggests that CAR may be upregulated both in levels and activation status prior to invasion where it plays a role in enabling tumor cell escape into the stroma. Our data further suggest that this may be enhanced in tumors with stiffer extracellular matrix environments, as it has been shown for other proteins such as Programmed Death-Ligand 1 (PD-L1) (50). Whilst the precise mechanisms by which CAR levels and phosphorylation are controlled by mechanical properties of the matrix remain unclear, this does point towards a bi-directional interplay between integrins (as key membrane associated force-sensing molecules) and CAR in the control of tumor cell invasion.

Our identification of CAR in complex with several key focal adhesion proteins would indicate CAR-dependent adhesion and migration may operate through direct co-operation with integrin-associated proteins. We additionally demonstrated a dependence upon phosphorylation of the CAR cytoplasmic tail for formation of these complexes. However, CAR is not clearly or robustly localised to focal adhesions at the basal surface of adherent cells, either in CMT 167 or LLC cell lines. This suggests that this complex may form at cell-cell junctions or within intracellular endocytic compartments. Indeed, FAK and Src have been shown to localise to cell-cell contacts in other cell types and immuno-precipitate with E-cadherin, α-catenin and β-catenin (10, 51–53). Both α-catenin and β-catenin have been suggested as potential interacting partners of CAR and may therefore mediate formation of this multi-protein complex (54, 55). FAK, Src and paxillin also associate with integrins and recent work from our lab and others have shown that integrins localise to cell-cell contacts in several epithelial-like cells (32, 56, 57). Moreover, we show that Src activity can help to promote CAR stability at cell-cell adhesion sites, indicating formation of this complex may in turn control CAR localisation. Given that our data show CAR forms a complex with total β1 integrins, we postulate that FAK, Src and paxillin may be recruited to cell junctions by CAR *via* β1 integrins to regulate cell-cell adhesion, as well as controlling levels of active integrins at the cell-matrix interface. It would be interesting to investigate traffic of integrins in live cells, given that recent evidence also suggests integrins can remain as active signalling proteins in endocytic compartments in cancer cells (58).

Our data show that Rap1 mediates CAR-dependent increase in cell-ECM adhesion and that CAR over-expression increases Rap1 activity. Rap1 has been suggested to induce integrin-mediated cell-ECM adhesion through enhanced integrin avidity and affinity for extracellular ligands, possibly *via* Rap1-GTP Interacting Adaptor Molecule (RIAM)-mediated recruitment of talin to integrin complexes (42). Other junctional molecules such as JAM-A, Nectin and E-cadherin can also regulate Rap1 activity, and dimerised JAM-A can induce Rap1 activation by bringing Afadin and PDZ-Guanine Exchange Factor 2 (GEF2) in the same complex (41, 59–61). VE-cadherin-induced Rap1 activation was shown to occur *via* Membrane Associated Guanylate kinase with Inverted orientation 1 (MAGI-1) which localises to cell-cell junctions and forms a complex with PDZ-GEF2 (62). It is possible that CAR regulates Rap1 activity by creating a complex with MAGI-1 and PDZ-GEF2, and similar to JAM-A, this complex could be formed *via* dimerization of CAR molecules and binding of partners through CAR PDZ-binding domains. Taken together, these data suggest that CAR could facilitate the formation of a complex between the Rap GEF PDZ-GEF2 and the scaffold protein MAGI-1 to activate Rap1 and regulate β1 integrin activity and cell adhesion.

In summary, we have identified a new role for CAR in mediating cell-matrix adhesion and invasion of lung cancer cells through co-operation with integrin-based adhesions and mechanosensing. Our work paves the way for future studies to explore the relationship between organisation of the extracellular matrix and CAR levels/phosphorylation in human tumors to determine whether this receptor represents a potential therapeutic target to prevent lung cancer cell metastasis.

## Data Availability Statement

The original contributions presented in the study are included in the article/**Supplementary Material**. Further inquiries can be directed to the corresponding author.

## Ethics Statement

The animal study was reviewed and approved by Ethical Review Committee at King’s College London and the Home Office, UK.

## Author Contributions

CO, MP, GS, and EO-Z all designed the research. CO, MP, and EO-Z analyzed data. CO, EP, JK, and EO-Z performed research. CO and MP wrote the paper. All authors contributed to the article and approved the submitted version.

## Funding

CO was supported by the National Institute for Health Research (NIHR) Biomedical Research Centre (BRC) based at Guy’s and St Thomas’ NHS Foundation Trust and King’s College London. EO-Z was supported by funding from the Medical Research Council UK (MR/S009191/1, to MP).

## Author Disclaimer

The views expressed are those of the author(s) and not necessarily those of the NHS, the NIHR or the Department of Health and Social Care.

## Conflict of Interest

The authors declare that the research was conducted in the absence of any commercial or financial relationships that could be construed as a potential conflict of interest.

## Publisher’s Note

All claims expressed in this article are solely those of the authors and do not necessarily represent those of their affiliated organizations, or those of the publisher, the editors and the reviewers. Any product that may be evaluated in this article, or claim that may be made by its manufacturer, is not guaranteed or endorsed by the publisher.

## Supplementary Material

Supplementary Figure 1 – Manipulation of CAR does not alter key EMT markers **(A)** Representative confocal images of specified cell lines fixed and stained for E-Cadherin (shown as single channel black and white, magenta in merged image) and DAPI (blue). GFP is shown in green in merged channel. Scale bars are 10 μm. **(B)** Quantification of E-cadherin intensity from line scans perpendicular to junctions in all cell lines from images as in **(A)**. Data from at least 30 junctions per cell line, representative of 3 independent experiments. **(C)** Western blots of lysates from indicated cell lines probed for E-cadherin, β-catenin and HSC70. Values shown beneath graph are from densitometry analysis of E-Cadherin blots normalised to parental cells from 4 independent experiments +/-SEM. **(D)** Western blots of lysates from indicated CMT cells probed for vimentin and HSC70. Representative of 4 independent experiments. **(E)** Representative confocal images of FRAP analysis of WTCAR-GFP vs AACAR-GFP over time. **(F)** Graph shows recovery curves of fluorescence intensity for each cell line from data as in **(E)**. Data is from at least 15 different ROIs per cell line, shown as mean +/-SEM, representative of 3 independent experiments. P values ***p < 0.0005, ****p < 0.0001.

## Abbreviations

Ad5FK, Adenovirus 5 FiberKnob; CAR, Coxsackie and Adenovirus Receptor; ECM, ExtraCellular Matrix; EMT, Epithelial-Mesenchymal Transition; FAK, Focal Adhesion Kinase; FRAP, Fluorescence Recovery After Photobleaching; GEF, Guanine Nucleotide-Exchange Factor; GFP, Green Fluorescent Protein; GST, Glutathione S-transferase; GTP, Guanine Triphosphate; HSC70, Heat Shock Cognate 70kDa; JAM, Junctional Adhesion Molecule; MAGI-1, Membrane Associated Guanylate kinase with Inverted orientation 1; PD-L1, Programmed Death-Ligand 1; PKC, Protein Kinase C; Rap1, Ras-related Protein 1; RIAM, Rap1-GTP Interacting Adaptor Molecule; ROCK, Rho-associated Protein Kinase; SCID, Severe Combined ImmunoDeficiency; ZO-1, Zonula Occludens 1.
