# Supplementary figures and images for: CAR Co-Operates With Integrins to Promote Lung Cancer Cell Adhesion and Invasion
Source: Front Oncol. 2022 Feb 14;12:829313. doi: 10.3389/fonc.2022.829313 (PMC8889575; doi:10.3389/fonc.2022.829313)

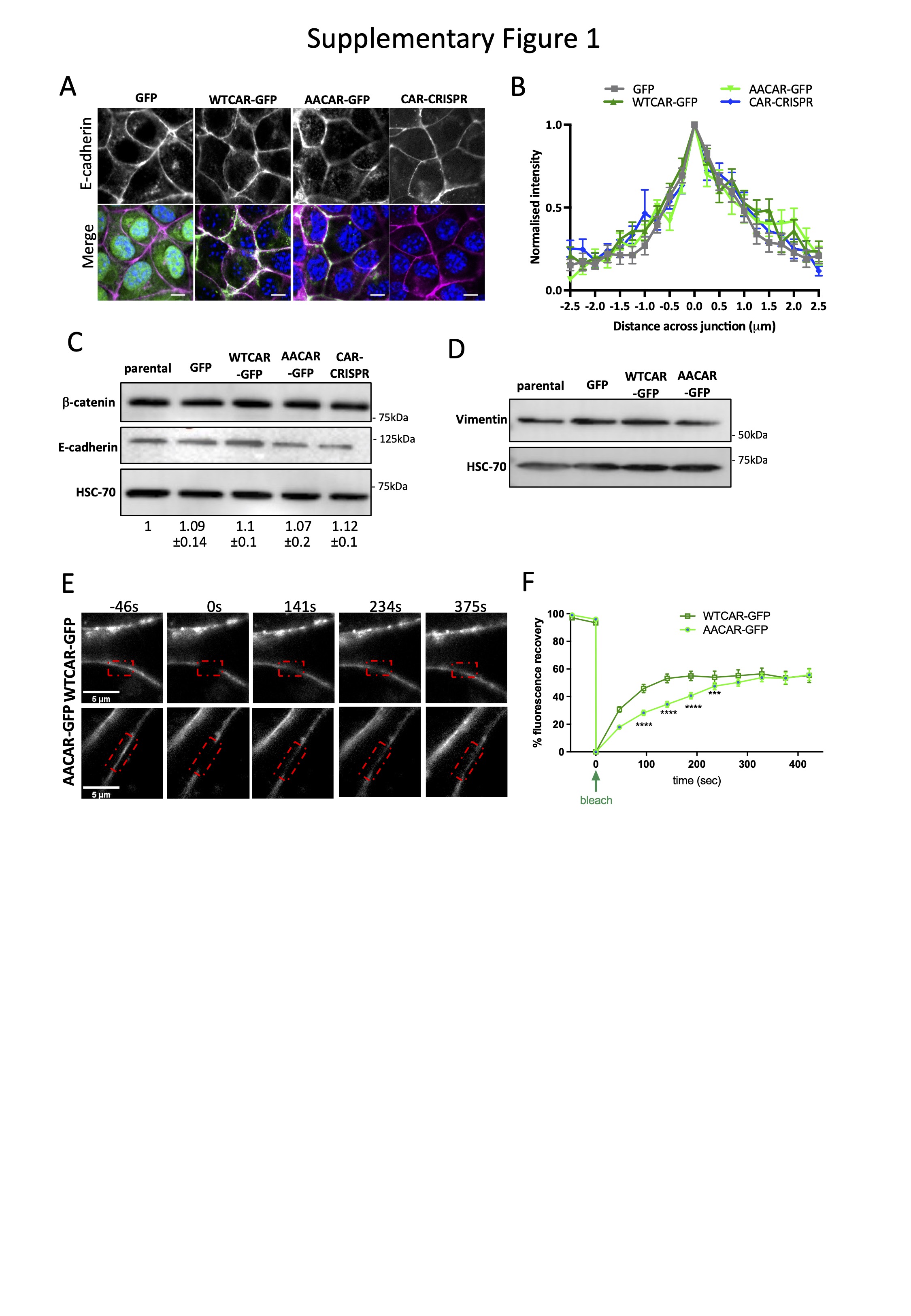

Supplement: Supplementary Figure 1 — – Manipulation of CAR does not alter key EMT markers (A) Representative confocal images of specified cell lines fixed and stained for E-Cadherin (shown as single channel black and white, magenta in merged image) and DAPI (blue). GFP is shown in green in merged channel. Scale bars are 10 μm. (B) Quantification of E-cadherin intensity from line scans perpendicular to junctions in all cell lines from images as in (A). Data from at least 30 junctions per cell line, representative of 3 independent experiments. (C) Western blots of lysates from indicated cell lines probed for E-cadherin, β-catenin and HSC70. Values shown beneath graph are from densitometry analysis of E-Cadherin blots normalised to parental cells from 4 independent experiments +/-SEM. (D) Western blots of lysates from indicated CMT cells probed for vimentin and HSC70. Representative of 4 independent experiments. (E) Representative confocal images of FRAP analysis of WTCAR-GFP vs AACAR-GFP over time. (F) Graph shows recovery curves of fluorescence intensity for each cell line from data as in (E). Data is from at least 15 different ROIs per cell line, shown as mean +/-SEM, representative of 3 independent experiments. P values ***p < 0.0005, ****p < 0.0001. [file Image_1.jpeg]
